# Applications and Safety of Sentinel Lymph Node Biopsy in Endometrial Cancer

**DOI:** 10.3390/jcm11216462

**Published:** 2022-10-31

**Authors:** Wan Kam Chiu, Shuk Tak Kwok, Yaokai Wang, Hiu Mei Luk, Aaron Hei Yin Chan, Ka Yu Tse

**Affiliations:** 1 Department of Obstetrics and Gynaecology, United Christian Hospital, Hong Kong; 2 Department of Obstetrics and Gynaecology, Queen Mary Hospital, The University of Hong Kong, Hong Kong; 3 Department of Obstetrics and Gynaecology, The University of Hong Kong Shenzhen Hospital, Shenzhen 518000, China

**Keywords:** endometrial cancer, sentinel lymph node biopsy, lymphadenectomy, micro-metastasis, isolated tumor cells, ultra-staging

## Abstract

Lymph node status is important in predicting the prognosis and guiding adjuvant treatment in endometrial cancer. However, previous studies showed that systematic lymphadenectomy conferred no therapeutic values in clinically early-stage endometrial cancer but might lead to substantial morbidity and impact on the quality of life of the patients. The sentinel lymph node is the first lymph node that tumor cells drain to, and sentinel lymph node biopsy has emerged as an acceptable alternative to full lymphadenectomy in both low-risk and high-risk endometrial cancer. Evidence has demonstrated a high detection rate, sensitivity and negative predictive value of sentinel lymph node biopsy. It can also reduce surgical morbidity and improve the detection of lymph node metastases compared with systematic lymphadenectomy. This review summarizes the current techniques of sentinel lymph node mapping, the applications and oncological outcomes of sentinel lymph node biopsy in low-risk and high-risk endometrial cancer, and the management of isolated tumor cells in sentinel lymph nodes. We also illustrate a revised sentinel lymph node biopsy algorithm and advocate to repeat the tracer injection and explore the presacral and paraaortic areas if sentinel lymph nodes are not found in the hemipelvis.

## 1. Introduction

Endometrial cancer (EC) is the most common gynaecological cancer in developed countries, and the incidence has been rising with aging and increased obesity of the population. In 2022, there will be an estimated 65,950 new cases and 12,550 deaths in the USA [1]. Surgery is the mainstay treatment for EC. Standard surgery includes total hysterectomy and bilateral salpingo-oophorectomy, with or without pelvic/para-aortic lymphadenectomy (LND) [2,3]. Lymph node (LN) assessment is important because LN metastasis is one of the most important prognostic factors for EC [4,5]. The 5-year overall survival (OS) for pelvic LN metastasis and para-aortic LN metastasis was found to be 57% and 49%, respectively [6]. The knowledge of LN status can also facilitate the use of adjuvant chemotherapy and radiotherapy to reduce the risk of distant and local recurrence [7,8]. However, the therapeutic value of LND has not been established. Two large randomized controlled trials (RCTs) in 2008 and 2009 showed that pelvic LND offered no therapeutic benefits compared with no LND in clinically early-stage EC [9,10]. A more recent multicenter retrospective study also demonstrated that LND had no survival benefit in an intermediate-risk group [11]. The Endometrial Cancer Lymphadenectomy (ECLAT) Trial is evaluating the survival effects of comprehensive LND in the absence of bulky nodes in patients with EC stages IB to II (all histological subtypes) and stage IA endometrioid International Federation of Gynecology and Obstetrics (FIGO) grade 3, serous, clear cell, or carcinosarcomas, and the results are expected in 2023 [12].

Sentinel lymph node (SLN) is the first LN reached by the metastasizing cells from the primary tumor before draining to the distal LNs [13]. In theory, if the SLN is negative, the remaining LNs in that lymphatic chain should also be negative. SLN biopsy (SLNB) is a minimally invasive technique used to identify the SLN and occult LN metastases. Nowadays, it replaces systematic LND in selected EC patients [2,3]. However, there are still myths about its usefulness in low-risk patients, and its safety in high-risk patients.

This review aims to review the roles of SLNB in EC, with a focus on its safety in high-risk patients and management of isolated tumor cells (ITCs) in the SLN.

## 2. Lymphatic Drainage of Endometrial Cancer

**Figure 1:**
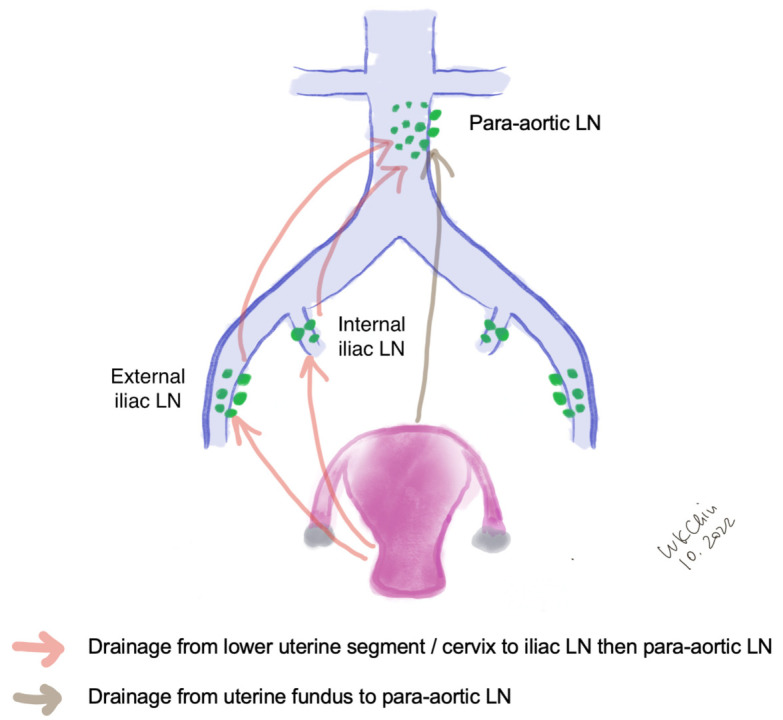
Lymphatic drainage of endometrial cancer. (LN, lymph nodes).

The lymphatic drainage of the endometrium is quite complex. The lymphatic drainage of the lower uterine segment is the same as that of the cervix, which drains through the parametria to the iliac and obturator nodes at the pelvic sidewall, common iliac LNs, para-aortic LNs and beyond (Figure 1). Alternative drainage near the uterine fundus develops along the gonadal vessels directly to the para-aortic nodes [14,15]. This implies that if the SLN is in the para-aortic region, it might be missed by the usual SLNB techniques that target the pelvic nodes (see Section 3). However, a prospective study of 742 patients reported that only 3% patients had isolated positive para-aortic nodes when pelvic nodes were negative [16].

## 3. Techniques of Sentinel Lymph Node Biopsy in Endometrial Cancer

Many factors such as age, depth of myometrial invasion and risk of lymphatic infiltration have been attributed to the successful rate of SLN mapping [17]. The performance of SLN biopsy is also affected by surgical expertise and the algorithm in SLN mapping failure [18].

### 3.1. Injection Sites

Different injection methods have been reported in the literature. Cervical injection is the most common approach. It had been shown that the cervical injection had a higher detection rate of pelvic SLN compared with the hysteroscopic injection at the endometrial tumor [19,20], while hysteroscopic injection had a slightly higher detection rate of para-aortic SLN especially isolated para-aortic LN compared to cervical injection [21]. This method is expensive because of the need for specialized equipment. Some reported that injection at dual sites (cervix and uterine fundal injection) increased the detection of SLN [22], and hysteroscopic injection and laparoscopic uterine fundal serosa injection also improved the para-aortic SLN detection [21,22]. Maramai et al. reported that in the case of failed bilateral mapping of SLN, cervical re-injection of ICG could significantly improve SLN detection rates from 73.3% to 94.5%, thus reducing the number of side-specific required lymphadenectomies [23].

To further improve SLN detection rate, some authors have combined preoperative lymphoscintigraphy with the injection of tracer. For example, Elisei et al. performed single-photon emission computerized tomography (SPECT) about three hours after Technetium (Tc)-99m-albumin cervical injection, and found that SPETCT could provide important anatomic information and enhance the intraoperative detection rate of SLNs [24]. At the same time, re-injection is another strategy that might improve the mapping rate of SLN in EC [23,25]. Cervical or hysteroscopic injections requires a long learning curve, and some studies suggested that an experienced physician was an important factor in improving the detection rate of SLN [25,26].

### 3.2. Injection Techniques and Tracers

**Figure 2:**
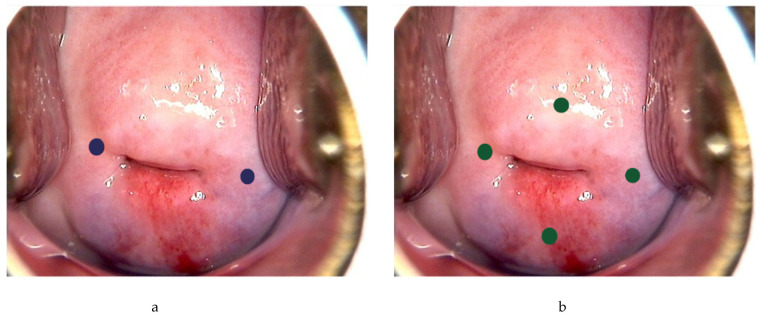
Injection of tracer at the cervix. Using ICG as example, 0.5–1 mL can be injected each superficially (1–3 mm at submucosa) and deeply (1–2 cm at stroma) at 3 and 9 o’ clock (blue dots) (**a**), or 0.5 mL each superficially and deeply at 3, 6, 9 and 12 o’clock of the cervix (green dots) (**b**).

Following general anesthesia, the tracer is injected into the submucosa (approximately 1–2 mm depth) and stroma (approximately 1–2 cm depth) of the cervix at 3 and 9 o’clock [21,23,24,25] (Figure 2a). According to the National Comprehensive Cancer Network (NCCN) guideline, injection at 3, 6, 9 and 12 o’clock of the cervix is another approach [3] (Figure 2b). The dye should be injected slowly at 5–10 sec per quadrant [27]. Retroperitoneal space needs to be opened. Retroperitoneal SLNs are then identified either by laparoscopic or open evaluation.

Colored dyes, including isosulfan blue and methylene blue, are affordable and they do not require special equipment. However, they have a low detection rate of SLN compared to other methods [28]. There is also a degree of subjectivity with the visual assessment [29]. Isosulfan blue was also associated a 1% risk of allergic reactions including anaphylaxis [26,27], and methylene had also been found to carry a small risk of paradoxical methemoglobinemia and serotonin syndrome [30], making them less favored nowadays.

Tc-99m is a metastable isomer that has become one of the most commonly used medical radioisotopes in diagnostic procedures since its introduction in the 1960s [31]. It has a half-life of about six hours, which can avoid excessive radio-exposure to the patients and the doctors. Tc-99m is injected at 3 o’clock and 9 o’clock of the cervix, and the signal is then identified intraoperatively by a gamma probe, with or without the SPECT–CT scan. Tc-99m has a better identification rate and sensitivity than methylene blue in many malignant tumors [32]. It can allow preoperative detection of SLN, and facilitate the detection of uncommon sites of nodal metastasis. The risk of allergic reactions is very rare with an estimated rate of 1-10/100,000 [33]. However, it requires special equipment and support from radiology departments. Besides, there is a small risk of radiation exposure to the medical personnel and the patients, thus limiting its widespread use in clinical practice.

Indocyanine green (ICG) is a water-soluble tricarbocyanine dye that penetrates tissues for up to 15 mm. It absorbs light at about 800 nm and emits light at about 830 nm [34]. The usual recommendation is to dilute the ICG to 0.5 mg/mL to 1.25 mg/mL using sterile water; 2–4 mL are to be injected [35]. The light emitted by the ICG is then visualized using a near-infrared fluorescent imaging system. It has good visibility and allows penetration of signal through intact tissue. Because of its safety and effectiveness, it has been widely used in hepatobiliary surgery, cardiac surgery, urology and other fields [36]. A meta-analysis showed that the detection rate of SLN was 77.8% with blue dye, 80.9% with Tc-99m, 86.3% with blue dye and Tc-99m, 92.4% with ICG alone, respectively, and up to 96.7% using ICG and blue dye based on two studies and 100% using ICG and Tc-99m based on one study [37]. The major disadvantage of ICG is the cost due to the requirement of the near-infrared fluorescent imaging system. In addition, ICG contains sodium iodine; there is an estimated 1/42,000 risk of anaphylactic reaction and iodine allergy is a contraindication to ICG [38]. The pooled sensitivity was above 90% regardless the choice of tracers. There was no significant difference in the detection rate between different surgical approaches [39]. As ICG has limited toxicity, higher bilateral detection rate and higher detection rate in obese patients especially with BMI >30, it has become more popular compared to other tracers [28,40,41].

The advantages and disadvantages of different methods are summarized in Table 1.

### 3.3. SLNB Algorithm

**Figure 3:**
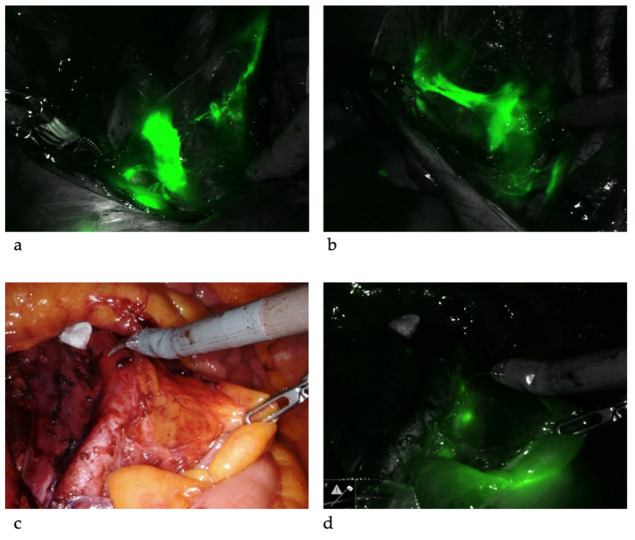
Sentinel lymph nodes. ICG was injected and SLNs were identified at left iliac (**a**) and right iliac (**b**) regions. When SLNs cannot be found in the pelvis, common iliac, presacral and para-aortic areas need to be explored. The picture illustrates a SLN at right common iliac near the presacral area (**c**,**d**).

**Figure 4:**
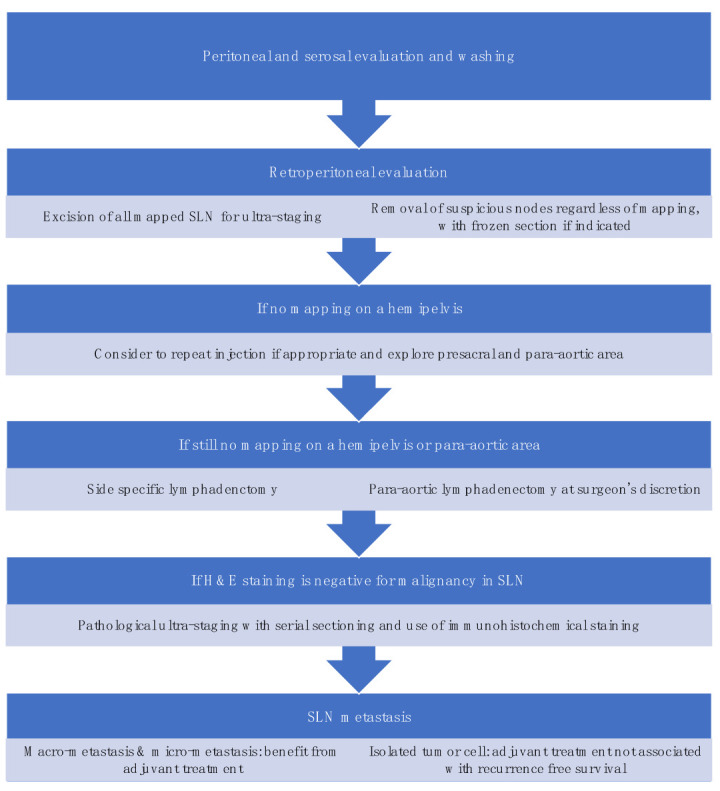
Sentinel lymph node biopsy algorithm. (modified from the NCCN guideline [3]).

SLNB algorithm refers to (1) peritoneal and serosal inspection and peritoneal washings; (2) retroperitoneal evaluation localization of stained lymphatic channels from the parametria to the primary nodal basin, and removing all SLNs and any suspicious nodes, with frozen section if indicated (Figure 3a); (3) retroperitoneal dissection up to common iliac region, presacral region and/or para-aortic region to look for rare isolated para-aortic LN especially when no pelvic SLN is found (Figure 3b); (4) a side-specific systematic LND if SLN is not detected on the ipsilateral hemipelvis [18,27,43] (Figure 4). It has been shown that the rate of systematic LND was reduced from 65% to 23% since the introduction of such SLNB algorithm [27].

Standardization in techniques and algorithms for SLNB is important in the diagnostic accuracy of SLNB which ultimately affects the oncological outcomes of the patients. Moloney et al. developed a surgical competency tool for SLNB in minimally invasive surgery (MIS) for EC, and made a consensus recommendation on the use of tracer, injection sites and technique, the dissection for SLN, and importantly, the troubleshooting in SLN mapping failure [44].

## 4. Safety of Sentinel Lymph Node Biopsy in Endometrial Cancer

EC is a heterogeneous disease composing of different histological subtypes and molecular alterations. It is often categorized into low-, intermediate-, and high-risk groups to predict the chance of metastasis and recurrence. However, the classification system is not standardized. The Cancer Genome Atlas (TCGA) project utilized various genomic, transcriptomic and proteomic analyses and classified EC into *DNA Polymerase Epsilon* (*POLE*), ultra-mutated (*POLEmut*), microsatellite instability hypermutated, copy-number low, and copy-number high [45]. The clinical behaviors vary a lot among these subtypes. The PORTEC 3 trial showed that patients with p53 abnormal (p53abn) EC had a poor prognosis, in contrast to patients with *POLEmut* EC who had excellent survival outcomes even with high-grade histology and advanced stage [46]. The European Society of Gynaecological Oncology (ESGO), the European Society for Radiotherapy and Oncology (ESTRO) and the European Society of Pathology (ESP) recently incorporated these molecular changes and defined different risk groups (Table 2) [2].

### 4.1. Early-Stage Low-Risk Endometrial Cancer

The prognostic value of assessing LN status in early-stage low-risk EC is controversial as the risk of LN involvement in this group is low. The incidence of pelvic LN metastasis and para-aortic LN metastasis for uterus-confined EC was 5–18% and 3–11%, respectively [47]. Considering the morbidity of systemic LND such as lymphedema, SLNB can provide prognostic information yet reduce the morbidities associated with systemic LND. One systemic review which included 18 studies suggested that the diagnostic accuracy of SLNB (pooled positive likelihood ratio (LR) 18.9 and negative LR 0.22) was superior to computed tomography (CT) (pooled positive LR 3.8 and negative LR 0.62) [48].

The SENTI-ENDO study, a prospective, multicenter cohort study, reported a sensitivity of 84% and negative predictive value (NPV) of 97% of SLNB in patients having stage I–II EC, where cervical injection of technetium and patent blue was used in SLNB followed by systematic LND [49]. Isolated para-aortic SLN was found in less than 1% in their cohort. Around 45% of women in this study had early-stage low-risk disease, including stage IA grade 1 or 2 type 1 EC. 11% of them were upstaged due to metastases in pelvic SLNs, while the other non-SLNs were all negative in final pathology [49]. With such high NPV and detection rate, SLNB can be considered as an alternative to systemic LND for LN assessment in early-stage low-risk EC [2].

MIS is now the standard route of operation for early-stage EC [18,50]. The FIRES study included 385 clinical stage I patients undergoing robotic surgery, among which 70% had grade 1–2 endometrioid adenocarcinoma, and reported a NPV of 99.6% and sensitivity of 97.2% using cervical injection of ICG [42]. A meta-analysis included eight studies on MIS in EC, and found that the overall detection rate of laparoscopic SLNB was 96% and the bilateral SLN detection rate was 73% [51]. Hence, SLNB is also an accurate tool in assessing LN status in MIS.

The oncological outcomes of SLNB in early-stage low risk EC were multifactorial, being influenced by the pathological evaluation of SLN, the use of adjuvant treatment and the surveillance after treatment. Enhanced pathological evaluation of SLN using ultra-staging and immunohistochemistry (IHC) staining could aid in detecting low volume LN metastasis. However, long-term survival data of SLNB is still lacking.

One retrospective multicenter study reviewed 304 patients having low- to intermediate-risk EC, and found 11.7% of patients with negative SLNs and 9% with positive SLNs, respectively [52]. The recurrence free survival (RFS) was not affected by SLN biopsy (hazard ratio (HR) 0.89, 95% confidence interval (CI) 0.42–1.90; *p = 0.77*). The SENTI-ENDO study showed the the 50-month RFS was 84.7% and SLNB did affect the use of adjuvant therapy [53]. Another retrospective study evaluated 223 patients with BMI 40.6 kg/m^2^ and it showed that there was no difference in 2-year OS rate (98% vs 98%; *p* = 0.7), disease-free survival (DFS) rate (99% vs 98%; *p* = 0.8)and (PFS) rate (97% vs 93%; *p* = 0.4) between patients with SLNB and systematic LND, respectively [54]. Currently the SELECT study [55] and ENDO-3 trial [56] are prospectively evaluating the oncological outcomes of early-stage patients undergoing SLNB.

### 4.2. Early-Stage High-Risk Endometrial Cancer

Most studies on SLNB were performed on early-stage low-risk EC patients and there has been no RCT on SLNB in high-risk patients. A systematic review included 9 prospective cohort studies on 429 stage I high-grade EC patients using cervical injection of ICG for SLN detection, where at least a bilateral pelvic LND was performed as the reference standard [57]. The pool detection rate per patient was 91%, the bilateral detection rate was 64%, the sensitivity was 92%, the false negative rate was 8%, and the negative predictive value was 97%. The findings were comparable with those studies on low-grade EC, and the false negative predictive rate was also similar to that observed in other cancer sites such as vulvar cancer [58], early stage breast cancer [59] and melanoma [60], where SLNB had already been established as the standard care. The performance in selected studies is illustrated in Table 3 [61,62,63]. Soliman et al. found only one patient (1%) with bilateral negative SLNs who was subsequently found to have metastatic non-SLN on the final pathology [62]. Cusimano et al. showed that 14 patients (52%) had metastatic disease in SLN only, and 7 (26%) were found outside the usual LND boundaries, implying that the LN metastases would not have been detected if SLNB was not performed [63].

The safety data of SLNB in high-risk patients are scarce. A retrospective study involving 6314 stage II EC patients showed that despite the reduced rate of systematic LND from 81.5% to 65.7% since the introduction of SLNB, the 3-year OS rates remained similar between patients undergoing SLNB and those having systematic LND (79.9% Vs 78.6%; HR 0.98, 95% Cl 0.80–1.20, *p* = 0.831) [64]. The OS was comparable in both groups in endometrioid and non-endometrioid subtypes. Compared to patients without surgical nodal evaluation, there was an OS by 34–44% in those having either LND or SLNB.

The Memorial Sloane Kettering Cancer Center (MSK) group reviewed 79 patients with SLNB and 166 undergoing systematic LND who had stage I – IV serous EC [65]. The 2-year PFS rate was 58.8% in the SLNB group and 64.9% in the LND group (*p* = 0.478), the 2-year OS rate was 89.1% and 83.9% (*p* = 0.9), and the recurrence rate was 36.7% and 40.9%, respectively. In either group, 60–80% recurred in peritoneum or distal organs. The authors then compared the survival after SLNB (performed in MSK) versus comprehensive pelvic and para-aortic LND (performed in the Mayo Clinic) in stage I–III serous and clear cell EC [66]. Patients with SLNB had a shorter inverse-probability of treatment weighting (IPTW)-adjusted 3-year recurrence-free survival (RFS) rate compared to those with systematic LND (69% Vs 80%; HR 1.46; 95% CI 0.70–3.04; *p = 0.32*), but their IPTW-adjusted 3-year OS rates were similar (88% Vs 77%; HR 0.44, 95% CI 0.19–1.02; *p* = 0.06).

The MSK also evaluated the survival outcomes of 136 patients with uterine carcinosarcoma undergoing SLNB [67]. Similarly, there was no difference between the SLNB and systematic LND groups, where the median PFS was 23 months Vs 23.2 months (*p* = 0.7), and the 2-year PFS rate was 38.7% and 47.6% (*p* = 0.5), respectively.

Adjuvant chemotherapy and radiotherapy could increase the 5-year RFS and OS rates of patients with p53abn EC by 22.4% and 23.1% [46]. As most patients with high-grade EC are p53abn and CTRT is suggested regardless of the stage of disease, the diagnostic or therapeutic benefit of SLNB becomes questionable [68]. On the other hand, SLNB may be more useful in p53 wildtype EC to ascertain the stage and determine the need for adjuvant therapy.

## 5. Management of SLN- and Non-SLN Metastasis

### 5.1. Pathological Ultra-Staging of SLNs

SLN mapping provides fewer nodes to be examined compared with traditional LND, thus allowing a more in-depth assessment of the sentinel nodes by the pathologists. Similar to breast cancer protocols, pathological ultra-staging of sentinel nodes is being utilized to detect previously undetectable nodal metastasis in EC [69].

Ultra-staging is performed if the initial hematoxylin and eosin (H&E) staining is negative for metastasis. There are different protocols for ultra-staging, most of which involve serial sectioning in 5 μm sections at each of two levels, 50-μm apart, and the use of IHC with anti-cytokeratin [70]. It can detect macro-metastasis (>2 mm), micro-metastasis (>0.2mm to ≤2 mm) and isolated tumor cells (ITC) (≤0.2 mm). The use of ultra-staging has resulted in higher detection of nodal involvement and stage migration from stage I/II to IIIC disease. It was estimated that >30% of stage IIIC EC might have been missed without ultra-staging [71]. This had subsequently increased the prescription of adjuvant treatment in patients who otherwise would not have been indicated.

### 5.2. Management of Micro-Metastasis and Isolated Tumor Cells in SLNs

The NCCN and ESGO/ESTRO/ESP guidelines advocated the use of adjuvant chemotherapy and/or external beam radiation therapy (EBRT) +/− vaginal brachytherapy in patients with stage IIIA to IIIC EC [2,3]. Adjuvant treatment comes with a side effect burden which can significantly affect quality of life. It is therefore worthwhile to determine the clinical significance of macro/micro-metastases and ITC to guide the need for further treatment.

It is widely accepted that patients with macro-metastasis benefit from adjuvant treatment [5,72]. However, the management for those with micro-metastasis (MM) and ITC, together termed low volume metastasis, is controversial and to date there are no definite international recommendations. There is evidence to suggest that patients with MM would also benefit from adjuvant treatment. A study comparing 428 patients, 70.6% had node-negative EC who did not receive adjuvant treatment, 22.2% had nodal MM and adjuvant treatment, and 7.2% had nodal MM without adjuvant treatment [73]. It was found that the second group (MM with adjuvant therapy) had similar DFS as the node-negative control (*p* = 0.648), but the third group (MM without adjuvant therapy) had significant reduced DFS (*p* = 0.0001). The use of adjuvant therapy in nodal MM patients could significant reduced the risk of recurrence or progression (HR 0.29, 95% CI 0.13–0.65).

The finding of ITC and its management is slightly more ambiguous. The incidence of ITC ranged from 2% to 36% [74,75,76], and was associated with deep myometrial invasion and tumor size [75]. A survey amongst gynecologic oncologists in Society of Gynecologic Oncology (SGO) in 2017 found that 21% would treat ITC as node positive disease [77]. A meta-analysis consisting of eight articles also found that 72% of patients with MM or ITC in SLNs received adjuvant therapies [78].

One retrospective study showed that the 3-year PFS rate was 95.5% in patients with ITC compared to 87.6% in those node-negative patients, and 32% of the former received no adjuvant therapy or just vault brachytherapy [74]. Another retrospective study including 844 patients found that the 3-year RFS was 86% in patients with ITC as compared with 90% in patients with negative nodes [79]. A more recent retrospective study compared the outcomes of 175 stage I–II endometrioid EC patients with ITC who underwent either adjuvant treatment (57%), or observation or vaginal brachytherapy only (43%) [80]. The recurrence rate was 4.6%, and multivariate analysis showed that the use of chemotherapy and external beam radiation (EBRT) were not associated with RFS. It was noteworthy that the choice of adjuvant therapy was based on other pathological risk factors like deep myometrial invasion and lympho-vascular invasion (LVSI). Lastly, the above meta-analysis showed that for patients with MM or ITC in SLNs who did not receive adjuvant therapy, the recurrence rate was similar to those without adjuvant therapy (relative risk (RR) 1.05, 95% CI 0.83–1.34; *p* = 0.979), and was also comparable to those node-negative patients who did not receive adjuvant treatment (RR 2.26, 95% CI 0.44–11.70; *p* = 0.072) [78].

Based on the above findings, it appeared that the presence of ITC alone should not be used as a sole prognostic marker to guide adjuvant therapy.

### 5.3. Non-SLN Metastasis

Metastasis in non-SLNs can occur up to 30–40% [81,82,83]. In theory, if non-SLNs are not sampled, occult nodal metastasis may be missed, which may lead to down-staging of the disease and omitting the use of adjuvant therapy which may otherwise be indicated. Therefore, it is important to learn if there are any predictive factors that can facilitate the identification of non-SLN metastasis.

Khoury-Collado et al. studied 266 patients with stage I–IV low- and high-risk EC undergoing SLNB, and found that the risk of metastasis in SLNs was almost 3 times higher than in non-SLNs (2.99% vs 1.11%; *p* = 0.0003) [84]. In the study by Touhami et al. which involved 266 stage I–II low- and high-risk EC patients, 34.8% were found to have non-SLN metastasis [81]. Multivariate analysis showed that size of SLN metastasis >2 mm was the only predictive factor for non-SLN metastasis (5% vs 60.8%; *p* < 0.0001), while histological type and grade, myometrial invasion, LVSI and cervical stromal invasion were not significant. Altin et al. analyzed 395 patients who had either SLNB or at least pelvic LND [83]. Among those 42 patients who had SLN metastasis, 16 patients had non-SLN metastasis. However, for patients with successful bilateral SLN mapping and negative SLN, none had non-SLN metastasis. Similar to Touhami’s findings, size of SLN metastasis was the only predictive risk factor for non-SLN metastasis, where 59%, 20%, 10% of patients with macro-metastasis, MM and ITC, respectively, were found to have non-SLN metastasis (*p* = 0.012), and multivariate analysis showed macro-metastasis had 8 times higher risk of non-SLN metastasis compared to low volume metastasis (OR 8.2, 95% CI 1.84–36.24; *p* = 0.006). In addition, for patients with SLN metastasis, none had non-SLN metastasis for those with low-risk disease, in contrast to 40% for those with intermediate-, high-intermediate- and high-risk EC.

Notably, back-up LND after SLNB could only increase the detection rate of nodal metastasis by 1% and it did not improve the DFS and OS of the patients [85].

## 6. Other Benefits of Sentinel Lymph Node Mapping

### 6.1. Intra-Operative Outcomes

There was no major difference between the intra-operative complication rates in both approaches (OR 0.48, 95% CI 0.20–1.17; *p* = 0.11) [86]. The conversion rate to laparotomy was also similar [87]. The estimated blood loss (EBL) after SLNB was between 20 mL and 160 mL [88]. A meta-analysis showed that SLNB resulted in less EBL than systematic LND (the mean difference −54.40, 95% CI −85.36 to −23.45; *p* < 0.001) [86]. One retrospective study that focused on patients with high BMI also revealed less EBL in SLNB patients than LND patients (30 mL vs 40 mL; *p* = 0.03) [54].

One meta-analysis reviewed 13 articles and found that the operating time of SLNB ranged from 118.8 min to 235 min [88]. A few retrospective studies reported that the operative time was reduced by 40–90 min in SLNB compared to LND [27,54,86,87,89,90].

### 6.2. Post-Operative Outcomes

Liu et al. reported a lower post-operative complication rate in laparoscopic SLNB compared to systematic LND (13% vs 5.2%, *p* = 0.04) [90]. Helgers et al. reviewed seven articles, and they also showed that patients with SLNB had fewer post-operative complications of any grade (OR 0.52, 95% CI 0.36–0.73; *p* = 0.0002) and post-operative complications of grade 3–5 (OR 0.52, 95% CI 0.28–0.96; *p* = 0.04), where the grades were assigned using the Clavien Dindo Classification [91]. However, another article found that the post-operative complication rates were similar between both methods, using a robot-assisted approach [87].

A few articles attempted to evaluate the post-operative complications in more detail. One retrospective study showed fewer patients requiring hospitalization for two days or more after SLNB compared to pelvic LND (IPTW adjusted 21.6% vs 7.8%; *p* < 0.001) [87]. However, another retrospective study found no difference between SLNB and LND (9.5 h vs 9.8 h; *p* = 0.81) [90]. The reoperation and readmission rates from the surgery were also similar between both approaches [87], though one article showed a higher readmission in patients undergoing LND compared to SLNB (4.6% vs 1.4%; *p* < 0.001) [92].

Based on the above results, the benefit of SLNB in the peri-operative outcomes compared to conventional LND appeared to be mild to moderate only. One possible reason was that over 80% of EC patients have hysterectomy and staging by MIS approach nowadays [93], where the incidence of peri-operative complications was already very low compared to laparotomy [94,95]. For example, it is impossible for SLNB to shorten the length of hospitalization further when the operations in EC can be done as a day-surgery in some centers [96,97,98].

### 6.3. Patient-Reported Outcomes

Lower extremity lymphedema (LEL) is the most common complication after LND and occurs in 4.6–47% of patients [99,100], and the risk can be related to the number of LNs dissected [101]. SLNB removed fewer LNs than systematic LND. So, it is legitimate to assume that there is less LEL. Geppert et al. reported that the incidence of LEL in the SLNB group was significantly lower than the systemic LND group (1.3% vs 18.1%; *p* = 0.0003) [102]. Leitao et al. conducted a study on patient-report LEL after SLNB, and the prevalence in SLNB and LND groups was 27% and 41%, respectively (OR 1.85, 95% CI 1.25–2.74; *p* = 0.002) [103]. After adjusting the BMI and the use of EBRT, LND remained a risk factor for LEL compared to SLNB (OR 1.8, 95% CI 1.22–2.69; *p* = 0.003).

The rate of lymphoceles was rarely reported. One study showed that patients undergoing SLNB compared to LND had lower rate of lymphoceles (2.6% vs 13.3%, *p* = 0.02) [102]. Diniz et al. reported similar rate of lymphocele (3.4% vs 14.1%; *p* = 0.009), and their multivariate analysis showed that systematic LND was the only independent risk factor for the development of lymphocele (OR 3.68, 95% CI 1.39–9.79; *p* = 0.009) [104]. To date there is no report on the incidence of lymphorrhea after SLNB, probably because the risk is very low (0–4%) after systematic LND [105,106].

## 7. Limitations of the Current Literature

Our review showed substantial heterogeneity in the study designs, SLNB protocols, injection sites and techniques, and ultra-staging techniques. Therefore, it is difficult to compare across different studies and perform meta-analyses. Earlier studies also focused on the detection performance of different SLNB techniques. Long-term oncological outcomes, including those patients who have low-volume metastases in SLNs, are yet to be determined. Besides, there is a lack of RCTs especially in high-risk EC. The introduction of the new molecular classification shifts the paradigm of management of EC, and the roles and effects of SLNB and other adjuvant therapies still await further research.

## 8. Conclusions

SLNB that follows a well-defined SLNB algorithm is an effective and accurate alternative to systematic LND for nodal assessment in clinical early-stage EC. It has high diagnostic accuracy, and it can also reduce the EBL, shorten the operative time, and lower the risk of LEL. More importantly, the survival outcome was similar compared with traditional LND. SLNB is now considered as a routine practice in early-stage low-risk EC. For early-stage high-risk EC, emerging evidence shows that SLNB is a reasonable alternative, though there is still a lack of RCTs. However, our review identified several gaps in the knowledge on SLNB and more research is needed.

## Figures and Tables

**Table 1:** Summary of the SLN detection rate with different injection sites and tracers. (ICG, indocyanine green; NA, not available; SLN, sentinel lymph node; SPECT, single-photon emission computerized tomography).

	SLN Detection Rate				Other Advantages/Disadvantages	
	Overall SLN	Pelvic SLN	Para-Aortic SLN	Isolated Para-Aortic SLN	Advantages	Disadvantages
Cervical (dye) [20]	89%	NA	NA	NA	Affordable No special instrument is required	Has a small risk of allergic reaction, paradoxical methemoglobinemia and serotonin syndrome Can be subjective in the visual assessment
Cervical (radioisotope) [20]	96%	NA	NA	NA	Can allow pre-operative detection of SLN with SPECT Can detect uncommon site of nodal metastasis Allergic reaction is very rare (1–10/100,000)	Requires support from radiology departments Requires gamma probe Exposure to radiation
Cervical (ICG) [19,21,42]	82–95.1%	77–78%	19.5–59%	0–6%	Limited toxicity Allergic reaction is very rare (1/42,000) No radiation exposure Good visibility	Expensive Requires near-infrared fluorescent imaging system
Hysteroscopic (radioisotope) [20]	78%	NA	NA	NA	Can allow pre-operative detection of SLN with SPECT Can detect uncommon site of nodal metastasis Allergic reaction is very rare (1–10/100,000)	Requires hysteroscopy Requires support from radiology departments Requires gamma probe Exposure to radiation
Hysteroscopic (ICG) [19,21,42]	33–82.6%	25–53%	25–29%	4–8%	Limited toxicity Allergic reaction is very rare (1/42,000) No radiation exposure Good visibility	Requires hysteroscopy Requires near-infrared fluorescent imaging system
Dual injection (cervical and fundal) with dual tracer (ICG and radioisotope) [22]	100%	98%	66.7%	NA	Good visibility High detection rate	Expensive Requires support from radiology departments Requires gamma probe Requires near-infrared fluorescent imaging system Exposure to radiation

**Table 2:** European Society of Gynaecological Oncology/European Society for Radiotherapy and Oncology/European Society of Pathology risk classification system of endometrial cancer and recommended lymph node evaluation methods. (modified from [2]).

Risk Group	Molecular Classification Unknown	Molecular Classification Known	Lymph Node Staging
Low	Stage IA endometrioid + low-grade + LVSI negative or focal	Stage I–II *POLEmut* endometrial carcinoma, no residual disease Stage IA MMRd/NSMP endometrioid carcinoma + low-grade + LVSI negative or focal	Sentinel lymph node biopsy
Intermediate	Stage IB endometrioid + low-grade + LVSI negative or focal Stage IA endometrioid + high-grade + LVSI negative or focal Stage IA non-endometrioid (serous, clear cell, undifferentiated carcinoma, carcinosarcoma, mixed) without myometrial invasion	Stage IB MMRd/NSMP endometrioid carcinoma + low-grade + LVSI negative or focal Stage IA MMRd/NSMP endometrioid carcinoma + high-grade + LVSI negative or focal Stage IA p53abn and/or non-endometrioid (serous, clear cell, undifferentiated carcinoma, carcinosarcoma, mixed) without myometrial invasion	Sentinel lymph node biopsy
High- intermediate	Stage I endometrioid + substantial LVSI regardless of grade and depth of invasion Stage IB endometrioid high-grade regardless of LVSI status Stage II	Stage I MMRd/NSMP endometrioid carcinoma + substantial LVSI regardless of grade and depth of invasion Stage IB MMRd/NSMP endometrioid carcinoma high-grade regardless of LVSI status Stage II MMRd/NSMP endometrioid carcinoma	Systematic lymphadenectomy Sentinel lymph node biopsy is an acceptable alternative in stage I/II
High	Stage III–IVA with no residual disease Stage I–IVA non-endometrioid (serous, clear cell, undifferentiated carcinoma, carcinosarcoma, mixed) with myometrial invasion, and with no residual disease	Stage III–IVA MMRd/NSMP endometrioid carcinoma with no residual disease Stage I–IVA p53abn endometrial carcinoma with myometrial invasion, with no residual disease Stage I–IVA NSMP/MMRd serous, undifferentiated carcinoma, carcinosarcoma with myometrial invasion, with no residual disease	Systematic lymphadenectomy Sentinel lymph node biopsy is an acceptable alternative in stage I/II Debulking of enlarged lymph nodes and para-aortic staging if pelvic lymph node involvement is found intra-operatively

**Table 3:** Summary of the performance of SLNB in high-risk endometrial cancer. (EC, endometrial cancer; ICG, indocyanine green; LND, lymphadenectomy; NA, not available).

Authors	Study Design	Number of Patients	Stage	High-Risk Features	Methods of Sentinel Lymph Node Biopsy	Detection Rate per Patient (%)	Unilateral Only Detection Rate (%)	Bilateral Detection Rate (%)	Sensitivity (%)	False Negative Rate (%)	Negative Predictive Value (%)
Persson J et. al. [55]	Prospective	257	I–II	Grade 3, or non-endometrioid histology, or deep myometrial invasion, or cervical stromal invasion, or a non-diploid cytometry	Cervical injection of ICG, followed by pelvic and infrarenal paraaortic LND	NA	NA	95	98	NA	99.5
Soliman PT et. al. [56]	Prospective	101	I–IV	Serous, clear cell, grade 3 endometrioid, or carcinosarcoma, biopsy proven cervical involvement or low-grade endometrioid tumors with radiologically suspected deep myometrial invasion	Cervical injection of tracers including ICG, blue dye or Tc-99m, followed by pelvic and para-aortic LND to the level of the renal vessels.	89	40	58	95	5	98.6
Cusimano MC [57]	Prospective	156	I	Grade 2 endometrioid or high-grade (grade 3 endometrioid, serous, carcinosarcoma, clear cell, undifferentiated or dedifferentiated, and mixed high-grade)	Cervical injection of ICG, followed by pelvic lymphadenectomy for grade 2 endometrioid EC, and pelvic and paraaortic LND for high-grade EC	97.4	99.9%	77.6	96	4	99
Schiavone MB [61]	Prospective	48	I–IV	Carcinosarcoma	Cervical injection of either blue dye with or without radiocolloid, or ICG	83	15	85	NA	NA	NA

## Data Availability

Not applicable.
